# Development of mental health first aid guidelines for suicidal ideation and behaviour: A Delphi study

**DOI:** 10.1186/1471-244X-8-17

**Published:** 2008-03-18

**Authors:** Claire M Kelly, Anthony F Jorm, Betty A Kitchener, Robyn L Langlands

**Affiliations:** 1 ORYGEN Research Centre, University of Melbourne, Australia

## Abstract

**Background:**

Suicide is a statistically rare event, but devastating to those left behind and one of the worst possible outcomes associated with mental illness. Although a friend, family member or co-worker may be the first person to notice that a person is highly distressed, few have the knowledge and skills required to assist. Simple guidelines may help such a person to encourage a suicidal individual to seek professional help or decide against suicide.

**Methods:**

This research was conducted using the Delphi methodology, a method of reaching consensus in a panel of experts. Experts recruited to the panels included 22 professionals, 10 people who had been suicidal in the past and 6 carers of people who had been suicidal in the past. Statements about how to assist someone who is thinking about suicide were sourced through a systematic search of both professional and lay literature. The guidelines were written using the items most consistently endorsed by all three panels.

**Results:**

Of 114 statements presented to the panels, 30 were accepted. These statements were used to develop the guidelines appended to this paper.

**Conclusion:**

There are a number of actions which are considered to be useful for members of the public when they encounter someone who is experiencing suicidal thoughts or engaging in suicidal behaviour. These guidelines will be useful in revision of curricula of mental health first aid and suicide intervention training programs. They can also be used by members of the public who want immediate information about how to assist a suicidal person.

## Background

Many different approaches have been tried to prevent suicide, but few have any strong supporting evidence. A systematic review of suicide prevention strategies concluded that education of physicians and restriction of lethal means were effective, while methods such public education, screening programs and media education need further evaluation [1]. Major reasons for the lack of evidence are the difficulty in researching the prevention of a statistically rare event like suicide and the ethical dilemma in withholding intervention in controlled trials. Despite these difficulties, there is much that can be done to improve the quality of interventions that are being tried.

In this paper, we aim to improve one particular approach to public education – training of members of the public in how to give first aid to someone who is suicidal. Two existing approaches of this sort are Applied Suicide Intervention Skills Program (ASIST) and Mental Health First Aid training.

ASIST [2] was initially developed at the University of Calgary, Canada, and is used in many parts of the world. The program focuses on identifying risk of suicide, ensuring short-term safety and connecting suicidal individuals with professional help, crisis care and informal support in the community. The program draws on the epidemiological and clinical risk factors for suicide and has been reported to be successful in many settings, however, formal evaluation of the program on a large scale has not been conducted.

Another program of this sort is Mental Health First Aid training [3] which was developed to train members of the public to assist others in getting appropriate professional help for mental disorders or assist in mental health crisis situations. When the program was first in development, the authors used evidence-based information wherever possible, but very little research was found about how members of the public, with no clinical training, could assist a friend, family member or acquaintance who was showing signs of mental disorder or crisis. For advice on how to manage these situations, the authors informally sought the opinions of clinical experts.

In order for these approaches to be effective, they need to ensure that the first aid strategies that are taught are likely to be helpful. Because controlled trials of component first aid strategies are not feasible, an alternative is to use expert consensus to develop a set of guidelines on strategies that are most likely to work. Such guidelines can be used directly as a source of advice by members of the public and they can inform the content of first aid training courses aiming to prevent suicide. The aim of this project was to develop such guidelines. These guidelines needed to focus on the immediate prevention of suicide, and not on solving the problems which lead to the crisis. The first aider's role would be to ensure the suicidal individual's safety until the crisis has passed or the person has chosen to seek appropriate professional help.

We chose the Delphi method, a technique used for reaching consensus in a group of experts or across expert groups. Our aim was to get consensus within and between panels of professionals, carers and consumers, so that the guidelines would be respectful of the needs of all three groups. This method is relatively inexpensive and simple to conduct, and can be done on the Internet. By conducting the research online, it was possible to include participants from English-speaking countries across the world, inexpensively and without lengthy postal delays. The Delphi methodology has been used in health research in the past, mainly to reach consensus amongst medical practitioners, but also with consumers of health services in some settings [4,5]. We have also successfully used this method to develop mental health first aid guidelines for depression and psychosis using panels of professionals, consumers and carers [6,7]. No research using the Delphi methodology to determine consensus on suicide first aid guidelines has been conducted previously.

## Methods

**Figure 1:**
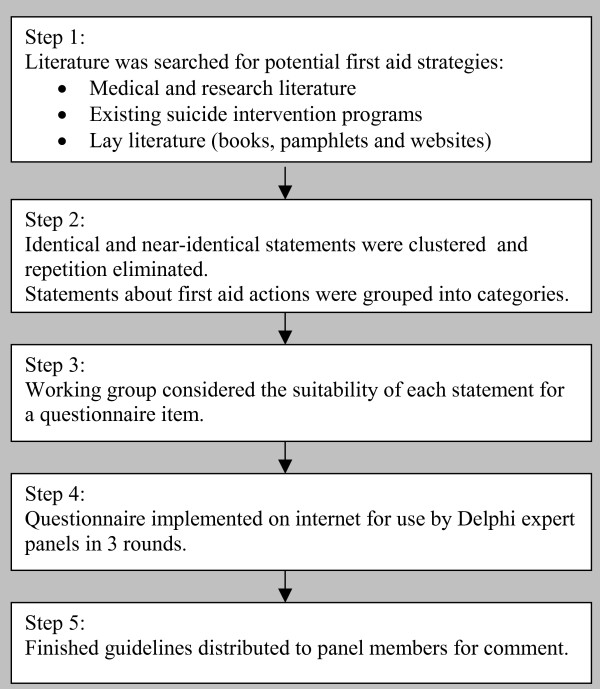
Stages in guideline development.

This study had two phases: a literature search and questionnaire development, and the Delphi process. Please see Figure 1 for a summary of the steps.

### Literature search

The aim of the literature search was to find statements which instruct the reader on how to determine whether someone is having thoughts of suicide, how to offer assistance in the short term, and how to access appropriate professional help for a suicidal person. The literature search was conducted across three domains: the medical and research literature, the content of existing suicide prevention and intervention programs, and lay literature. The lay literature included books written for the general public, particularly carers' guides, websites and pamphlets.

The medical and research literature was accessed through searches of PsycInfo and PubMed. The search term was 'suicide' and all records for the 20 years leading to the search date were reviewed. The search term 'suicide' generated far too many records, but all attempts to narrow the search were found to be unsatisfactory as they excluded too many relevant records. Papers which described assessment of suicide risk, brief suicide interventions, or guidelines for treating suicidal patients were reviewed, a total of 234 papers. While much of the advice given in these papers was considered too clinically orientated to be useful for first aid, a number of papers did include brief advice and simple intervention instructions. Statements were drawn from 42 of the 234 relevant records. All statements felt to be simple enough for lay people to use were included.

To find appropriate websites, we used the search engines Google [8], Altavista [9], and Yahoo [10] using the search term 'suicide'; the first 50 websites listed by each were reviewed; beyond the first 50 websites, quality declined rapidly. Since most websites were listed by more than one search engine, only 68 websites were reviewed. The websites were read thoroughly, once again looking for statements which suggested a potential first aid action (what the first aider should do) or relevant awareness statement (what the first aider should know). Any external links to other websites were followed and the same process applied to each of them.

The fifty most popular books on the Amazon [11] website which listed the word 'suicide' in the title or keywords were selected. This site was chosen because of its extensive coverage of books in and out of print, including works about mental health aimed at the public. Books which were autobiographical in nature, self-help guides and clinical manuals were excluded. The remaining books were read to find useful statements. The majority of these were carers' guides, which do contain advice relevant for first aid.

Any relevant pamphlets were sought and read, and statements were taken from these as well. The majority of the pamphlets were written and distributed by organisations focussing on mental health in general, or suicide in particular, but some were more general community organisations. Most of these pamphlets were obtained from websites, but where these were not available online, a request was made for relevant materials from large mental health and community organisations. While the majority of the lay literature focussed on understanding suicide, supporting people who have lost a loved one to suicide, and being aware of the risk factors and warning signs which might indicate that someone was thinking about suicide, there was also some advice to friends and family members on what to do if they are concerned that someone they love may be at risk.

Where available, the training materials from existing courses which address suicide intervention were also reviewed and statements were drawn from these. Only a small number of training courses were found to be relevant, as the majority of such training is developed for professionals with previous clinical training in specific settings. The courses for which material was reviewed were the existing Mental Health First Aid Program [12], the Applied Suicide Intervention Skills Training (ASIST) Program (LivingWorks Canada) [2,13], and the Mental Illness First Aid Course (Canadian Mental Health Association) [14].

### Questionnaire development

The questionnaire was developed by first grouping statements into categories: identification of suicide risk, assessing seriousness of the suicide risk, initial assistance, talking with a suicidal person, no-suicide contracts, ensuring safety, confidentiality, and passing time during the crisis. Similar or near-identical statements were frequently derived from multiple sources, and they were not repeated in the questionnaire. A working group comprised of the authors of this paper and colleagues working on similar projects convened at each stage of the process to discuss each item in the questionnaire. The role of the working group was to ensure that the questionnaire did not include ambiguity, repetition, items containing more than one idea or other problems which might impede comprehension. The wording was carefully designed to be as clear, unambiguous and action-oriented as possible. For example, 'the first aider should find out if the person is thinking about harming themselves' is better stated 'the first aider should ask the person if they have been having thoughts of suicide'. All statements were written as an instruction as shown in the above example. The only items which were not included in the questionnaire were those which were so ambiguous that the working party was not able to agree on the meaning of the statement, or those which called upon 'intuition' or 'common sense', as these cannot be taught.

The majority of participants answered the questionnaire via the Internet, using an online survey website, Surveymaker [15]. Three participants requested paper copies of the questionnaire, as they did not have convenient access to the Internet. Participants were able to stop filling in their questionnaires at any time and log back in to continue, without the risk of losing the completed section of their questionnaires. Using the Internet also made it very easy for the researchers to identify those who were late in completing questionnaires and send reminders, with no need to send extra copies of the questionnaire. No questions were inadvertently missed, as the web survey was set up so that each question was mandatory. In addition, such survey software allows for branching, so participants who did not endorse the use of no-suicide contracts were not asked to answer questions about what such a contract should contain.

### Statement selection

The criteria for item inclusion in the questionnaire have been articulated above; items which are non-clinical in nature, interpretable by the research team, teachable and useful to a member of the public with only minimal training were included. To clarify, consider the examples below.

"Consider a brief hospitalisation for your suicidal patient."

While a professional such as a family doctor or psychiatrist may recommend hospitalisation, a member of the public could not take this action themselves. This item is very clinical in nature, and not useful to a member of the public, so was not included.

"You need to walk the walk with the person you are helping."

This statement cannot be interpreted literally, so was not included.

"It is important to follow your instincts when helping a suicidal person."

This statement asks the reader to draw on instinct, which cannot be taught or effectively described. It was not included in the questionnaire.

"If you suspect that someone may be suicidal, you should ask them directly."

This item is clear and concise, will lead to accurate identification of the problem, and can be done by anyone, so it was included in the questionnaire.

### The Delphi process

Participants were recruited into one of three panels: professionals (clinicians and researchers), consumers (people who had experienced suicidal ideation or a suicide attempt in the past) and carers. The professional panel had 22 experts, the consumer panel 10, and the carer panel 6. All panel members were from developed English speaking countries (Australia, New Zealand, The United States, England and Canada). Participants were recruited in a number of ways. Professionals recruited were those who had publications in the areas of suicide intervention or prevention, identification of suicidal ideation, or treatment of suicidal patients. When letters were sent to professionals asking them to be involved, they were also invited to nominate any colleagues who they felt would be appropriate panel members. Those active in clinical practice were also asked to consider any former patients who might be willing to be involved. The 22 professional participants included 5 psychologists, 5 psychiatrists, 3 managers of mental health services, 2 social workers, 1 nurse, 9 researchers and 3 professors of psychology. Some participants had multiple roles in research, teaching and clinical work. Consumers were recruited from advocacy organisations, and referral by clinicians. They were also identified if they had written websites offering support and information to other consumers, or published memoirs. Carers were recruited through carers' organisations, but were difficult to recruit for this study. It may be that few carers see themselves as being adequately experienced in dealing with suicide crises, having been involved in perhaps only one. In some cases they may not be aware that the person they care for has been suicidal or even made a suicide attempt in the past. We discussed approaching support groups for people who had lost a loved one to suicide, but it was decided this would be inappropriate and may be distressing to the people in the groups.

Three rounds of questionnaires were distributed as follows, with each statement being rated up to two times. In round 1 the questionnaire, derived from the process described above, was given to the panel members. The questionnaire included space after each of the sections to add any suggestions for new statements that panel members felt should be included.

In each round of the study, the usefulness of each statement for inclusion in the mental health first aid guidelines was rated as *essential*, *important*, *don't know *or *depends*, *unimportant*, or *should not be included*. The options *don't know *and *depends *were collapsed into one point on the scale because operationally, they are the same response; most of the statements were, very reasonably, noted to be useful in some cases and not others, meaning they could not be generalised in guidelines, which is also true of statements participants did not feel confident to rate.

The suggestions made by the panel members in the first round were reviewed by the working group and used to construct new items for the second round. Although the carers' ratings were not used in the final analysis, their suggestions in round 1 for new ideas were included in round 2. Suggestions were accepted and added to round 2 if they represented a truly new idea, could be interpreted unambiguously by the working group, and were actions. Suggestions were rejected if they were near-duplicates of items in the questionnaire, if they were too specific (for example, "I get my husband to do some woodwork"), too general ("just be there"), or were more appropriate to therapy than first aid ("develop a strategy for coping with intense emotions, specific to the emotion; depression, anger, guilt").

Items rated as *essential *or *important *by 80% or more the professional and consumer panels were accepted for inclusion in the guidelines. If they were endorsed by 80% or more of one of the panels, or by 70–80% of both panels, they were re-rated in the subsequent round. Items which met neither condition were rejected. Before the second and third rounds of the study, each participant was sent a summary of the results of the previous round, listing which items had been accepted, which had been rejected, and which were to be re-rated. When an item was to be re-rated by the panellists, they were provided with their own response and a table outlining how many people in each group had endorsed the item. They were told that they did not have to change their responses when re-rating an item, but that if they wished to, they would have the opportunity to do so.

## Results

Table 1 shows the continuity of participation across the three rounds. By the third round, no carers remained engaged with the process, and their earlier responses were not included in the final results.

**Table 1:** Study participation in each round

	**Round**	**Round**	**Round**
**Group**	**1**	**2**	**3**
Consumers	10	9	7
Carers	6	3	0
Professionals	22	18	15

**Figure 2:**
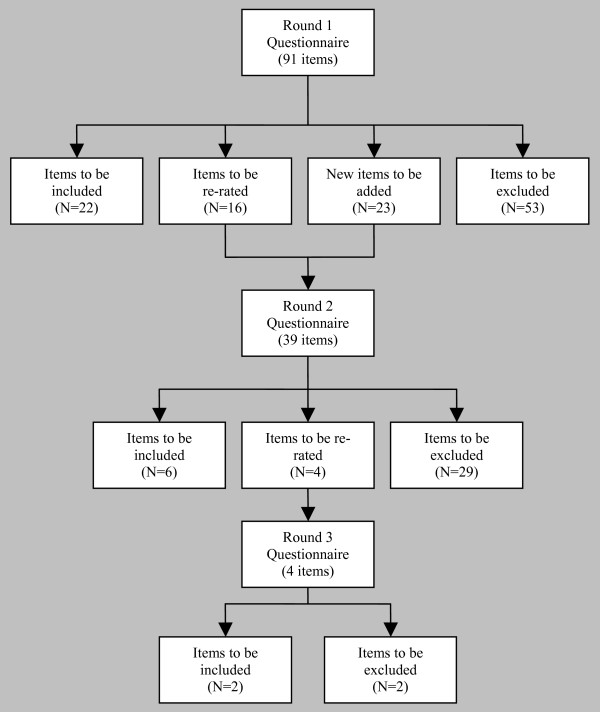
Items accepted, rejected and re-rated at each round.

Figure 2 shows the rates of acceptance, rejection, and re-rating of the items in each round of the questionnaire. Of the 91 items included in the first round, 22 were accepted, 53 were rejected, and 16 met criteria for re-rating. An additional 23 new items were created from suggestions made by the panellists. Of the 39 items included in the questionnaire for the second round, 6 were accepted, 29 were rejected, and 4 met criteria for re-rating. In the second round, there was no option to suggest new items for the third round. Of the 4 items included in the questionnaire for the third round, 2 were accepted and 2 were rejected. Of the total of 114 statements rated by the panels, 30 were accepted. (See Table 2 for a categorised list of accepted items, and Additional file 2 for a complete list of every item including the number of professionals and consumers endorsing each item).

**Table 2:** Statements accepted as mental health first aid guidelines

**Item:**	**Round:**
**Section 1: Identification of suicide risk**	
If the first aider thinks someone might be having suicidal thoughts, they should ask that person directly.	1
The first aider should be aware that if a person is not suicidal, asking them will not the idea of suicide in their head.	1
The first aider should not avoid using the word 'suicide'. It is important to discuss the issue directly, without dread or expressing negative judgement.	1
The first aider should understand that the threat of suicide may indicate that a person is trying to communicate to the first aider how badly he or she feels.	1
If the first aider appears confident in the face of a suicide crisis, it may have a reassuring effect for the suicidal person.	1
The first aider should allow the suicidal person to discuss their feelings. A suicidal person may feel relief at being able to do so.	1
If the first aider clearly states that thoughts of suicide may be associated with a treatable disorder, this may instil a sense of hope for the suicidal person.	2
The first aider should be able to recognise the warning signs of suicide.	2
**Section 2: assessing seriousness of the suicide risk**	
The first aider should establish whether the person has definite plans and intentions to take his life as opposed to vague suicidal notions such as "what's the point," or "I can't be bothered going on".	1
The first aider should ask the suicidal person if they have a plan for suicide.	1
The first aider should ask the suicidal person how they intend to suicide.	1
The first aider should find out if the suicidal person has already taken steps to secure the means to end their life.	1
The first aider should ask the suicidal person if they have decided when they will carry out their plan.	1
The first aider should ask the suicidal person if they have been using drugs or alcohol.	1
The first aider should ask the suicidal person if they have ever made a suicide attempt in the past.	1
The first aider should take all thoughts of suicide seriously. The lack of a plan for suicide is not sufficient to ensure safety.	3
**Section 3: initial assistance.**	
The first aider must not leave someone who is feeling suicidal on their own.	1
The first aider must keep in mind that they may not be successful in preventing suicide.	3
**Section 4: Talking with a suicidal person**	
The first aider should tell the suicidal person that they care and want to help.	1
The first aider needs to allow the suicidal person to talk about their reasons for wanting to die.	1
The first aider should remind the suicidal person that these thoughts need not be acted on.	1
Suicidal thoughts are often a plea for help and a desperate attempt to escape from problems and distressing feelings. The first aider should therefore allow the suicidal person to talk about those feelings.	1
By discussing specific problems, the first aider can help the person work out ways of dealing with the difficulties that seem insurmountable.	1
The first aider needs to find out what has supported the suicidal person in the past, and whether these supports are still available.	1
The first aider should encourage the suicidal person to do most of the talking.	2
The first aider should express empathy for the suicidal person.	2
**Section 5: No-suicide contracts**	
The first aider should try to develop a contract with the suicidal person to ensure their safety.	1
Contracts should include 24 hour safety contacts in case the suicidal person feels unable to continue with the agreement not to attempt suicide (such as a suicide helpline, professional helper or family member).	1
**Section 6: Ensuring safety**	
*No items endorsed*.	
**Section 7: Confidentiality**	
The suicidal person needs to be involved in decisions about who else knows about the suicidal crisis.	2
A first aider must never agree to keep the suicidal person's suicidal plans a secret.	2
**Section 8: Passing time during the crisis**	
*No items endorsed*.	

### Writing the Guidelines

It was important to the research team to avoid making the guidelines read like a list of 'dos' and 'don'ts'. The accepted items were incorporated into a plain language document. To illustrate, consider the following statements:

1. If the first aider thinks someone might be having suicidal thoughts, they should ask that person directly.

2. The first aider should not avoid using the word 'suicide'. It is important to discuss the issue directly, without dread or expressing negative judgement.

These statements were incorporated to make the following paragraph:

If you suspect someone may be at risk of suicide, it is important to ask them directly about suicidal thoughts. Do not avoid using the word 'suicide'. It is important to ask the question without dread, and without expressing a negative judgement. The question must be direct and to the point. For example, you could ask: "are you having thoughts of suicide?" or "are you thinking about killing yourself?"

The guidelines were developed from these accepted statements, however, there were some difficulties. The major issue was that while professionals and carers strongly endorsed statements which directed the first aider to seek professional help while the person was suicidal, the consumers did not. Correspondence from consumers on the panel indicated that the crisis care they had received in hospitals was often punitive, and might include being placed in physical restraints, spoken to unkindly, and told they were wasting the hospital's time. Carers, on the other hand, felt ill-equipped to cope with a suicidal friend or family member and preferred to seek help whenever such a crisis arose. It is worth noting here that not all of the consumers rejected the idea of professional help; only items endorsed by 80% or more of each panel were accepted and half of the consumers did endorse professional help as important.

The only professional help item accepted by the whole panel was an item stating that if a person is suicidal they should be provided with safety contacts, including professional help. An item was also accepted advising the person to consider utilising supports they had used in the past if these were still available. Such supports might include professional help, but might not. In addition, panelists accepted a number of statements which relate to risk assessment, such as the extent to which a plan for suicide has been developed and the means for suicide accessed, and whether the person has made a previous suicide attempt. While these risk factors would help a professional to shape an intervention, they are not sufficient for a first aider to prevent suicide, and are left hanging.

To address this discrepancy, we included a paragraph in the guidelines explaining that professionals think that professional help should always be sought, but that some people who have been suicidal disagree. We acknowledged that carers were often torn between wanting to seek help for the suicidal person and being afraid of alienating them. We did not say that help should or should not be sought, but we gave enough information to allow the first aider to make an informed decision.

When the guidelines were in draft form, they were sent to all the panel members for feedback, along with an explanation of the professional help issues we encountered. We asked panel members to respond to the addition, whether they supported it, wanted it removed, or had a suggestion of how it could be improved. Overall, the response supported the inclusion of the paragraph but, as we expected, a small number of panellists disagreed. No consumer panellists objected to the inclusion, but some of the professionals felt that the guidelines needed to state categorically that professional help must be sought. The paragraph remained as written.

For the rest of the guidelines, only feedback related to readability and structure was sought and incorporated. The guidelines are appended to this article and can be freely distributed (see Additional file 1).

## Discussion

In spite of differing opinions between consumers, carers and professionals about when it is appropriate to seek professional help, we have demonstrated that it is possible to reach consensus on first aid strategies for suicidal thoughts and behaviours. These guidelines can be used as a source of advice to the public in their own right, as a basis for determining the curriculum of first aid training courses, and as a standard against which to evaluate the quality of existing materials and programs. Although the guidelines were designed for the public, they may also contain advice that might be helpful to people working in health and welfare professions.

While the aim of this project was to find statements which were acceptable to everyone, two major themes were brought to light when we considered the items which were rejected because of differences between the three panels; for example, those statements accepted by the carers, but not by consumers. These themes were the right of consumers to refuse help and the need for carers to preserve the life of the person they are helping, whatever it takes. Professionals' responses did not strongly support one theme or the other.

### Consumers' priorities: understanding, not professional help

A strong theme was the right of consumers to refuse help. A number of items which were highly endorsed by both the clinicians and the carers were rejected by the consumers. The item which read "If it is known that the person is suicidal, the first aider must call a doctor, psychiatrist, or other professional" was endorsed by all of the carers and 72% of the professionals, but only 13% of the consumers. In addition, the item which read "If the first aider clearly states that thoughts of suicide may be associated with a treatable disorder, this may instil a sense of hope for the person" was endorsed by over 80% of the professional and carer panels, but only by half of the consumer panel. This suggests that some consumers disagree with a psychopathology-related explanation of suicidal thoughts and the idea that professional help is needed.

All of the carers and professionals said that if there was any risk to the first aider – because the suicidal person was becoming aggressive or brandishing a weapon – that the first aider should not try to intervene, but call emergency services for assistance. Only 60% of the consumers agreed. This again seems to suggest that for the consumers, the right to refuse assistance was of high priority.

Consumers felt it was important to be able to discuss their feelings and then make their own decision; 88% of consumers endorsed the item "The first aider should accept the suicidal feelings for what they are and discuss suicide as a possibility rather than an unthinkable act". Only half of the carers and professionals endorsed this item. While it would be hard for someone motivated to preserve life to listen to someone talk about suicide as a possibility, it may be that a suicidal person feels relief and understanding at being able to discuss their thoughts.

### Carers' priorities: preserve life at any cost

Carers were more likely than consumers and professionals to endorse items which protect the life of the suicidal person at any cost. An item which read "If the first aider can't get the suicidal person to hand over the means of suicide, emergency services must be contacted" was endorsed by over 80% of carers, but less than half of the professionals and only a quarter of the consumers. Over 80% of carers felt it was important to get professional help if the suicidal person was intoxicated, a reasonable action given the number of suicides which involve the use of drugs and alcohol. However, only a quarter of the consumers and half of the clinicians agreed.

The carers in this group were often looking after more than one family member with a mental illness and most had experienced more than one suicide-related crisis in the past. Only 5 carers completed the first round of the study (one completed a little more than half), 3 completed the second round and none completed the third round, although 10 carers had initially agreed to take part. During the study period, 2 carers informed the senior author that there had been further suicide attempts made by the people they care for during that time. These carers had a great deal of experience helping someone who is suicidal, and perhaps this experience explains why they often endorsed items which related to actively seeking the assistance of a professional helper. However, they also had a great deal of responsibility, often working outside the home, running a household and caring for one or more family members with mental illness, which may explain why they were not able to complete the study.

### Limitations

One limitation of this study is the small number of panel members, particularly in the carers' panel. It is important as well to reiterate that all panellists were recruited from developed English-speaking countries, so that the guidelines may not be generalisable to other countries or to minority cultures within those countries. Furthermore, these guidelines cannot stand alone, as they do not address the underlying psychological distress or mental illness which causes an individual to become suicidal. They need to be used in conjunction with the other guidelines in this series, including first aid for depression, first aid for psychosis, and first aid for non-suicidal self-injury [6,7]. These other guidelines can be downloaded from the Mental Health First Aid website [16].

## Conclusion

This process has proven that it is possible to develop guidelines for suicide first aid which are acceptable to professionals, people who have been suicidal, and to carers. Where the guidelines are used as the basis for first aid training, efforts need to be made to evaluate their impact on the first aiders' helping behaviours and on the recipients of the first aid, as far as this is possible. This will assist researchers to develop an evidence base for mental health first aid and suicide prevention initiatives.

## Competing interests

CMK is a Master Trainer of the Applied Suicide Intervention Skills Training course, developed by LivingWorks Canada. The remaining authors have no competing interests to declare.

## Authors' contributions

CMK and AFJ prepared the manuscript. All authors reviewed the manuscript. AFJ and BAK developed the methodology. CMK did the literature searches and wrote the first draft of the questionnaire. All authors contributed to the development of later versions of the questionnaire. CMK wrote the attached guidelines. All authors reviewed and suggested improvements to the guidelines.

## Pre-publication history

The pre-publication history for this paper can be accessed here:



## Supplementary Material

Additional file 2 **2 spreadsheets**. Spreadsheet 1: All items, categories, rates of endorsement and final outcomes. Spreadsheet 2: List of categories, number of items in each and outcomes at each round. Click here for file

Additional file 1 **First aid guidelines for suicidal thoughts and behaviours**. This file may be distributed freely, with the authorship and copyright details intact. Please do not alter the text or remove the authorship and copyright details. Click here for file
