# Supplementary material for: Development of mental health first aid guidelines for suicidal ideation and behaviour: A Delphi study
Source: BMC Psychiatry. 2008 Mar 18;8:17. doi: 10.1186/1471-244X-8-17 (PMC2324091; doi:10.1186/1471-244X-8-17)
Supplement: Additional file 1 — First aid guidelines for suicidal thoughts and behaviours. This file may be distributed freely, with the authorship and copyright details intact. Please do not alter the text or remove the authorship and copyright details. [file 1471-244X-8-17-S1.pdf]

SUICIDAL THOUGHTS & BEHAVIOURS
FIRST AID GUIDELINES

MENTAL HEALTH FIRST AID

Purpose of these Guidelines

These guidelines are designed to help members of the public to provide first aid to someone who is at risk of suicide. The role of the first aider is to assist the person until appropriate professional help is received or the crisis resolves.

Development of these Guidelines

The following guidelines are based on the expert opinions of a panel of mental health consumers, carers and clinicians from Australia, New Zealand, the UK, the USA and Canada about how to help someone who may be at risk of suicide.
Details of the methodology can be found in: (…)

Although these guidelines are copyright, they can be freely reproduced for non-profit purposes provided the source is acknowledged.
Enquiries should be sent to: Professor Tony Jorm, ORYGEN Research Centre, Locked Bag 10, Parkville, VIC 3052, Australia,
email: ajorm@unimelb.edu.au.

How to use these Guidelines

These guidelines are a general set of recommendations about how you can help someone who may be at risk of suicide. Each individual is unique and it is important to tailor your support to that person's needs. These recommendations therefore may not be appropriate for every person who may be at risk of suicide. Also, the guidelines are designed to be suitable for providing first aid in developed English-speaking countries. They may not be suitable for other cultural groups or for countries with different health systems.

An important note:

Self-injury can indicate a number of different things. Someone who is hurting themselves may be at risk of suicide. Others engage in a pattern of self-injury over weeks, months or years and are not necessarily suicidal. These guidelines can assist you only if the person you are helping is suicidal. If the person you are assisting is injuring themselves, but is not suicidal, please refer to the guidelines entitled First aid for deliberate self-injury.:

How can I tell if someone is feeling suicidal?

It is important that you know the warning signs of suicide.

Signs a person may be suicidal:

- Threatening to hurt or kill themselves
- Looking for ways to kill themselves: seeking access to pills, weapons, or other means
- Talking or writing about death, dying or suicide
- Hopelessness
- Rage, anger, seeking revenge
- Acting recklessly or engaging in risky activities, seemingly without thinking
- Feeling trapped, like there's no way out
- Increasing alcohol or drug use
- Withdrawing from friends, family or society
- Anxiety, agitation, unable to sleep or sleeping all the time
- Dramatic changes in mood
- No reason for living, no sense of purpose in life

Adapted from Rudd et al (2006).
Warning signs for suicide: Theory, research and clinical applications. Suicide and Life-Threatening Behavior, 36:255-262

3857_07 A4 VERSION ORYGENKJ

People may show one or many of these signs, and some may show signs not on this list.

If you suspect someone may be at risk of suicide, it is important to ask them directly about suicidal thoughts. Do not avoid using the word 'suicide'. It is important to ask the question without dread, and without expressing a negative judgement. The question must be direct and to the point. For example, you could ask:

- "Are you having thoughts of suicide?" or
- "Are you thinking about killing yourself?"

If you appear confident in the face of the suicide crisis, this can be reassuring for the suicidal person.

Although some people think that talking about suicide can put the idea in the person's mind, this is not true. Another myth is that someone who talks about suicide isn't really serious. Remember that talking about suicide may be a way for the person to indicate just how badly they are feeling.

How should I talk with someone who is suicidal?

It is important to:

- Tell the suicidal person that you care and that you want to help them.
- Express empathy for the person and what they are going through.
- Clearly state that thoughts of suicide are often associated with a treatable mental disorder, as this may instil a sense of hope for the person.
- Tell the person that thoughts of suicide are common and do not have to be acted on.

Suicidal thoughts are often a plea for help and a desperate attempt to escape from problems and distressing feelings. You should encourage the suicidal person to do most of the talking, if they are able to. They need the opportunity to talk about their feelings and their reasons for wanting to die and may feel great relief at being able to do this.

It may be helpful to talk about some of the specific problems the person is experiencing. Discuss ways to deal with problems which seem impossible to cope with, but do not attempt to 'solve' the problems yourself.

How can I tell if the situation is serious?

First, you need to determine whether the person has definite intentions to take their life, or whether they have been having more vague suicidal thoughts such as 'what's the point of going on?'. To do this, you need to ask the person if they have a plan for suicide. The three questions you need to ask are:

1. Have you decided how you would kill yourself?
2. Have you decided when you would do it?
3. Have you taken any steps to secure the things you would need to carry out your plan?

A higher level of planning indicates a more serious risk. However, you must remember that the absence of a plan is not enough to ensure the person's safety. All thoughts of suicide must be taken seriously.

Next, you need to know about the following extra risk factors:

- Has the person been using alcohol or other drugs? The use of alcohol and other drugs can make a person more susceptible to acting on impulse.
- Has the person made a suicide attempt in the past? A previous suicide attempt makes a person more likely to make a future suicide attempt or to kill themselves.

Once you have established that the risk of suicide is present, you need to take action to keep the person safe.

How can I keep the person safe?

A person who is actively suicidal should not be left on their own. If you can't stay with them, you need to arrange for someone else to do so. In addition give the person a safety contact which is available at all times (such as a telephone help line, a friend or family member who has agreed to help, or a professional help giver).

It is important to help the suicidal person to think about people or things that have supported them in the past and find out if these supports are still available. These might include a doctor, psychologist or other mental health worker, family member or friend, or a community group such as a club or church.

Do not use guilt and threats to prevent suicide. For example, do not tell the person they will go to hell if they die by suicide, or that they will ruin people's lives by killing themselves.

What about professional help?

During the crisis

Mental health professionals advocate always asking for professional help, especially if the person is psychotic. If the suicidal person has a weapon or is behaving aggressively towards you, you must seek assistance from the police in order to protect yourself.

However, the person you are helping may be very reluctant to involve a professional and, if the person is close to you, you may be concerned about alienating them. In fact, some people who have experienced suicidal thoughts or who have made plans for suicide feel that professional help is not always necessary.

After the crisis has passed

After the suicide crisis has passed, ensure the person gets whatever psychological and medical help they need. Other guides in this series may be useful for you in achieving this.

What if the person makes me promise not to tell anyone else?

You should never agree to keep a plan for suicide a secret. However, you should respect the person's right to privacy and involve them in decisions regarding who else knows about their suicidal intentions.

The person I am trying to help has injured themselves, but insists they are not suicidal. What should I do?

Some people injure themselves for reasons other than suicide. This may be to relieve unbearable anguish, to stop feeling numb, or other reasons. This can be distressing to see. There are guidelines in this series entitled First aid guidelines for deliberate self-injury which can help you to understand and assist if this is occurring.

A final note

Do your best for the person you are trying to help.

Remember, though, that despite our best efforts, some people will still die by suicide.

The MHFA Training & Research Program
ORYGEN Research Centre
Department of Psychiatry The University of Melbourne AUSTRALIA

Professor Tony Jorm, ORYGEN Research Centre
Locked Bag 10, Parkville VIC 3052 Australia
email: ajorm@unimelb.edu.au

All MHFA guidelines can be downloaded from **www.mhfa.com.au**
